# Regulatory Frameworks for Clinical Trial Data Sharing: Scoping Review

**DOI:** 10.2196/33591

**Published:** 2022-05-04

**Authors:** Nachiket Gudi, Prashanthi Kamath, Trishnika Chakraborty, Anil G Jacob, Shradha S Parsekar, Suptendra Nath Sarbadhikari, Oommen John

**Affiliations:** 1 The George Institute for Global Health New Delhi India; 2 Public Health Evidence South Asia, Prasanna School of Public Health, Manipal Academy of Higher Education Manipal India; 3 The George Institute for Global Health, University of New South Wales New Delhi India; 4 Prasanna School of Public Health, Manipal Academy of Higher Education Manipal India

**Keywords:** clinical trial, data sharing, policy, scoping review

## Abstract

**Background:**

Although well recognized for its scientific value, data sharing from clinical trials remains limited. Steps toward harmonization and standardization are increasing in various pockets of the global scientific community. This issue has gained salience during the COVID-19 pandemic. Even for agencies willing to share data, data exclusivity practices complicate matters; strict regulations by funders affect this even further. Finally, many low- and middle-income countries (LMICs) have weaker institutional mechanisms. This complex of factors hampers research and rapid response during public health emergencies. This drew our attention to the need for a review of the regulatory landscape governing clinical trial data sharing.

**Objective:**

This review seeks to identify regulatory frameworks and policies that govern clinical trial data sharing and explore key elements of data-sharing mechanisms as outlined in existing regulatory documents. Following from, and based on, this empirical analysis of gaps in existing policy frameworks, we aimed to suggest focal areas for policy interventions on a systematic basis to facilitate clinical trial data sharing.

**Methods:**

We followed the JBI scoping review approach. Our review covered electronic databases and relevant gray literature through a targeted web search. We included records (all publication types, except for conference abstracts) available in English that describe clinical trial data–sharing policies, guidelines, or standard operating procedures. Data extraction was performed independently by 2 authors, and findings were summarized using a narrative synthesis approach.

**Results:**

We identified 4 articles and 13 policy documents; none originated from LMICs. Most (11/17, 65%) of the clinical trial agencies mandated a data-sharing agreement; 47% (8/17) of these policies required informed consent by trial participants; and 71% (12/17) outlined requirements for a data-sharing proposal review committee. Data-sharing policies have, a priori, milestone-based timelines when clinical trial data can be shared. We classify clinical trial agencies as following either controlled- or open-access data-sharing models. Incentives to promote data sharing and distinctions between mandated requirements and supportive requirements for informed consent during the data-sharing process remain gray areas, needing explication. To augment participant privacy and confidentiality, a neutral institutional mechanism to oversee dissemination of information from the appropriate data sets and more policy interventions led by LMICs to facilitate data sharing are strongly recommended.

**Conclusions:**

Our review outlines the immediate need for developing a pragmatic data-sharing mechanism that aims to improve research and innovations as well as facilitate cross-border collaborations. Although a *one-policy-fits-all* approach would not account for regional and subnational legislation, we suggest that a focus on key elements of data-sharing mechanisms can be used to inform the development of flexible yet comprehensive data-sharing policies so that institutional mechanisms rather than disparate efforts guide data generation, which is the foundation of all scientific endeavor.

## Introduction

### Background

Data sharing from clinical trials is a contested space; it has gained salience particularly during the COVID-19 pandemic. Clinical data are defined as “the data, results, information, discoveries, inventions, processes and methods (whether patentable or not) resulting from or developed by investigator or study personnel in the performance of the clinical trial, but excludes all personal information and medical records” [1]. Clinical trial data sharing is defined as *“*sharing of anonymized, patient-level clinical trial data through established platforms thereby enhancing transparency, thus maximizing value of research and creating opportunities for external researchers to reanalyze, synthesize, replicate, and build upon previous evidence*”* [2,3]. Sharing anonymized individual participant data (IPD) along with other trial-generated data can often pave ways for informed clinical decisions. In particular, the secondary analysis of such clinical trial data helps in building on the body of existing evidence by consolidating data across smaller, underpowered studies. It is one of several cost-effective measures for augmenting a body of evidence in resource-constrained settings and in health emergencies [4]. The COVID-19 pandemic is considered a booster for clinical data sharing because many researchers and working groups have strongly advocated it [5-8]. Ideally, clinical trial data sharing needs to be harmonized and standardized for the global scientific community. However, to align with the purpose of research, data from human participants should benefit others, and data sharing is one of the best ways to achieve this.

Clinical trial agencies have provided guidelines for regulating data sharing in clinical research. In particular, the International Committee of Medical Journal Editors [9], the UK-based Pragmatic Clinical Trials Unit [10], and the US-based National Institutes of Health (NIH) StrokeNet [11] have developed guidelines for efficiently sharing and accessing data. Clinical trial registries and scholarly publications expect biomedical researchers to provide statements on sharing data during various stages of clinical trials (eg, at the time of trial registration, after the planned interim analysis, at the midterm, and at the end of the clinical trial), as applicable according to the respective data-sharing guidelines. These data-sharing guidelines aim to safeguard the privacy of study participants when data are used by a researcher to build on existing evidence (secondary research) and thereby maximize benefits for the public [12]. According to the clinical trial registration policy of the International Committee of Medical Journal Editors, there are prerequisites for data oversight or the presence of an institutional ethics committee to abide by the Good Clinical Practice guideline as outlined by the International Council for Harmonisation [13]. These prerequisites need to be operationalized through gaining the informed consent of study participants to ensure the safety of the study participants, investigators [13,14], and those involved in clinical trials. Furthermore, data sharing from clinical research is generally governed by national regulatory agencies in their respective locations [14,15].

Where such guidance exists, regulatory policy documents provide guidance on data sharing and access to data by ensuring participant safety and ethical compliance [16]. The emerging conflicts between data-sharing practices and potential threats to the privacy and confidentiality of trial participants are significant challenges faced by investigators in complying with data-sharing principles. The review of data-sharing guidelines by Blasimme et al [17] and a stakeholder consultation of 1329 scientists [18] demonstrated that the importance of data sharing in medical science is not sufficiently recognized. Technical, legal, and ethical barriers hinder data sharing from clinical trials. Technical barriers include lack of standardization, limited researcher capacity to build high-quality data, and a lack of financial incentives for data sharing. Legal barriers such as intellectual property rights (IPR), data ownership, concerns of data provider and data user regarding mutual benefits, and explicit informed consent for data sharing are threats to data sharing [3,5,17,18]. Similar concerns were shared by the public in a high-income setting where widely shared data could be a risk for patient privacy and could give rise to discrimination and exploitation [19].

### A Complicated Issue

The competing interests of stakeholders involved in clinical trials make data sharing a complicated issue owing to factors relating to investments and existing legal frameworks surrounding IPR. The resistance from for-profit pharmaceutical corporations is also understandable when they advocate data exclusivity, given their financial investments in conducting clinical trials. Most large multicenter clinical trials are funded by for-profit pharmaceutical corporations. Besides being data generators, these corporations are investors and risk takers, as well as intellectual conceptualizers of complex scientific information. Not surprisingly, such corporations have the incentive, control, and power to restrict data sharing. The agreement on Trade-Related Aspects of Intellectual Property Rights at the World Trade Organization (WTO) identifies transparency and availability of the latest information as being pivotal to trade and policy [20-22]. An updated list of IPR measures specific to a region and country limits the control exercised by multinational corporations [23]. Concerns over clinical trial data sharing follow IPR-related issues that arise with the sharing of undisclosed trial information—a practice often referred to as “secret trial data” [24,25]. For-profit pharmaceutical corporations often resist, or lack interest in, data-sharing efforts through their data-exclusivity practices [26,27]. This can have, and has had, a negative impact on access to medicines and biologicals, including vaccines in low- and middle-income countries (LMICs) [28].

Institutional frameworks based on jurisdictions vary considerably. Data sharing from cross-border or multicountry and multisite randomized controlled clinical trials are generally not governed by a single (or even comparable) national legislation [29,30]. Although international regulations on data sharing are lacking, there are a few guidelines. In a multicenter and multicountry trial, there are context-specific issues such as the cost of trial completion and data sharing, subcontracting, and the use of third parties to complete the trial. Nuances in subcontracting the conduct of the trial to for-profit and not-for-profit organizations further make data sharing difficult. There are ethical complexities as well: large sample sizes are often possible only by recruiting participants from low-income countries owing to their larger populations and (often) poor clinical trial oversight and regulatory mechanisms [19,31]. Not surprisingly, therefore, the wider acceptance of data-sharing practices in the absence of a mandate to share clinical trial data has created uncertainties among clinical trial investigators [32,33]. With the paucity of surveys or academic syntheses to offer guidance on data sharing, it is necessary to collate evidence and classify this information to facilitate syntheses and comparability with regard to data-sharing practices.

Given the limitations in the existing landscape of clinical trials regarding data sharing, it must be noted that at the institutional level, systematic steps are being taken to shift data sharing in more institutionalized directions, which is laudable. This is based on disclosures by funders; nevertheless, such disclosures vary in degrees. For ease of comprehension, we view these in the binary categories of open- or controlled-access models of sharing data. Pursuant to this, trial investigators are implementing data sharing according to varied milestones, depending on the progress of the clinical trial. Thus, at the aggregate level, timelines for disclosures also vary. In brief, such principal investigators connect the level of data disclosure to the completion of varied milestones. Our review appropriately classifies this information.

Considering the aforementioned gaps, this review attempts to synthesize the existing state of practices around clinical trial data sharing. Our viewpoint is decidedly from a public health perspective because we believe that data sharing needs to be promoted for the public good. With this intention, we conducted a scoping review of the literature with the following objectives: to identify regulatory documents that have guided clinical trial investigators in trial data sharing and to explore the key elements of data-sharing mechanisms in these regulatory documents.

## Methods

### Overview

A scoping review approach facilitates an understanding of emerging evidence and is often considered the first step in research evidence development [34]. We followed the JBI methodology, as proposed in the methodological framework of Arksey and O’Malley [35] for scoping studies and the work on advancing this methodology by Levac et al [36]. The review protocol was developed a priori; however, because of the time-bound nature of this review, we could neither register nor publish the protocol. The JBI methodology has outlined six steps for the conduct of a scoping review: (1) identifying the research question; (2) identifying relevant studies; (3) study selection; (4) charting the data, (5) collating, summarizing, and reporting the results; and (6) stakeholder consultations [34-36]. These steps are further described in the following sections. The scoping review is reported according to the PRISMA-ScR (Preferred Reporting Items for Systematic Reviews and Meta-Analyses extension for Scoping Reviews) guidelines [37].

### Step 1: Identifying the Research Question

As we intended to synthesize a fast-growing but fragmented body of literature on regulatory documents for data sharing, we did not follow the typical *Population, Intervention, Comparison, and Outcomes* or *Population, Concept, and Context* approach to guide our article selection process because this topic falls beyond the scope of these and other review classifications [38]. We developed the objectives of this scoping review using an iterative approach. Stakeholders (a group of domain experts) from the data-sharing working group of the COVID-19 Clinical Research Coalition were involved in providing feedback on the objectives [39]. In particular, one of the authors (OJ) is a member of the data-sharing working group of the COVID-19 Clinical Research Coalition, and this review was undertaken as a specific deliverable with technical support from the data-sharing working group.

### Step 2: Identifying Relevant Studies

We followed a 2-pronged approach for identifying regulatory documents: (1) literature search in scientific journals and (2) gray literature search. We conducted searches on MEDLINE (PubMed), SCOPUS, CINAHL (EBSCO), EMBASE, ProQuest, and Google Scholar using the keywords *data sharing policy*, *data sharing guidance*, *clinical trial data sharing*, and *individual participant data sharing*. The search was carried out by 2 authors (NG and SSP) on May 11, 2021. A detailed search strategy for each database is presented in Multimedia Appendix 1.

An alert was created between May 11, 2021, and August 31, 2021, on the aforementioned databases for the search strategy to further include articles as and when published. This step was deemed necessary because the topic is dynamic and published scholarly evidence has been emerging regularly since the COVID-19 pandemic began. The gray literature is an important source for gathering further evidence on data sharing. To populate a comprehensive list of trial agencies, we manually looked at the trial websites through a Google search. We also searched for data-sharing policies on the clinical trial agency websites (Multimedia Appendix 2 [16,40-64]). We further conducted reference screening of articles included at the full-text stage to identify any potential inclusions. All search results were uploaded into EndNote software (Clarivate), and duplicates were removed.

### Step 3: Study Selection

The selection of studies was carried out by 2 authors (PK and TC) independently in 2 sequential stages, namely, title-abstract and full-text stages. We used this 2-stage strategy because the evidence suggests that there is no difference between the titles-first and title-abstract–together approaches [65]. Conflicts on study selection were discussed until consensus was reached, or a senior team member (NG or OJ) acted as an arbitrator to decide on final inclusion or exclusion of the record. To ensure transparency in the study selection process, the number of records included at each stage was represented in the PRISMA (Preferred Reporting Items for Systematic Reviews and Meta-Analyses) 2020 chart, along with the reasons for exclusion of studies during the full-text screening [66]. The study selection was based on the following inclusion and exclusion criteria:

- Inclusion criteria: as this is a broad and emerging topic, we did not limit our review to studies with any specific research designs. We included studies that described any clinical trial data–sharing policies or standard operating procedures, which are defined as detailed, written instructions to achieve uniformity of the performance of a specific function upholding the goal of the Good Clinical Practice guideline [67]. This may include but not be limited to evidence synthesis papers such as systematic reviews and rapid evidence synthesis. We also included commentaries, editorials, and policy briefs; however, we restricted our search to studies published in English.
- Exclusion criteria: we excluded conference abstracts because these are susceptible to changes (eg, content or title) at the completed-manuscript stage, making it challenging to locate them. As our search was comprehensive, our decision to exclude conference abstracts would have had minimal impact in terms of the number of articles retrieved at the title-abstract stage.

### Step 4: Charting the Data

Data coding was carried out independently by 2 authors (PK and TC) using a predesigned yet flexible data-coding template (Multimedia Appendix 3). The study team members were consulted before finalizing the data-coding template, and minor modifications were incorporated based on the team’s feedback. The data-coding template was pretested as suggested by Levac et al [36] and Daudt et al [68]. Extracted data were coded as per characteristics of the regulatory documents (name of trial agency, type of regulatory document, recent version, regulatory document and policy scope, country of origin, geographical scope, scientific scope, timeline, and grant limit) and data-sharing mechanism (need for data-sharing agreement, informed consent, type of review committee, timeline to share and access data, cost of data sharing, and data-sharing model). Clinical trial agencies were categorized as being either *for-profit pharmaceutical trial agencies*, *federal or national regulatory agencies* (publicly funded trial agencies), *academic institutions* (affiliated to universities), *nonprofit research organizations*, or *networks/consortia of clinical trial units*.

Depending on the description of the data access requirement, we coded the data access model as either an *open-access model* or a *controlled-access model*. If the anonymized trial data are made available to the public without submission of a proposal or without an approval process and no limitations or restrictions on data use, it was coded as an *open-access* model. If the data request is reviewed against prespecified criteria by internal or external review committees, we coded it as a *controlled-access* model [69]. Furthermore, if the information in the included documents was insufficient, we referred to the source (often websites) of the respective documents to elicit further information; there was 1 such policy document [40,70].

### Step 5: Collating, Summarizing, and Reporting the Results

We used a narrative synthesis approach to summarize findings to provide a comprehensive view of an emerging topic owing to its potentially large volume and heterogeneous nature. Critical appraisals of studies and summarizing results from individual studies were beyond the scope of this review.

### Step 6: Stakeholder Consultation

As we sought to code data from the available pool of scientific guidelines, global standards, and national policies, conducting a stakeholder consultation for summarizing the results was beyond the scope of this project. Stakeholders from the data-sharing working group of the COVID-19 Clinical Research Coalition were consulted for finalizing the review objectives as described in step 1.

## Results

We have presented the findings of this scoping review based on the evidence identified through a scientific literature search and a gray literature search.

### Findings From Scientific Literature Search

**Figure 1:**
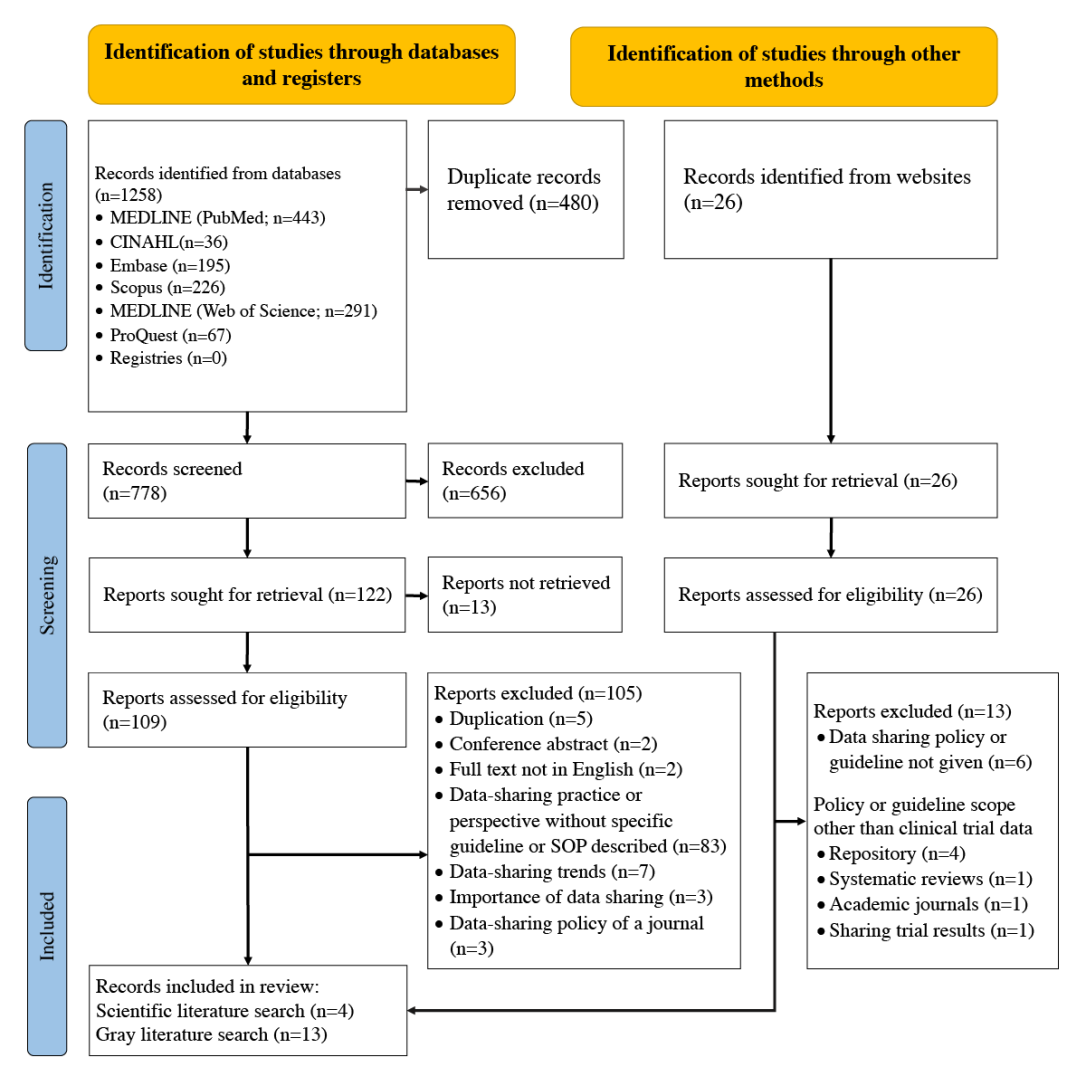
PRISMA (Preferred Reporting Items for Systematic Reviews and Meta-Analyses) 2020 chart. SOP: standard operating procedure.

The initial search yielded a total of 1258 records; after 480 (38.16%) duplicates were removed, we screened 778 (61.84%) titles and abstracts, and included 109 (8.66%) reports for the eligibility check. After the full-text screening of these 109 articles, we finally included 4 (3.7%) for the data coding [70-73]. Papers with a focus on trends in data sharing and importance of data sharing were excluded. The PRISMA 2020 chart is presented in Figure 1.

### Findings From Gray Literature Search

We found few academic publications on regulatory frameworks from clinical research. Therefore, we decided to conduct a gray literature search. During the full-text review stage, we made a note of the referred clinical trial agencies, and the websites of these agencies were examined for clinical trial data–sharing policies—either a policy, a standard operating procedure, or a guiding document on data sharing. We included a total of 13 [10,11,16,41-44,74-79] trial agencies that had at least one data-sharing policy or guiding document. A list of trial agencies can be found in Table 1. We have provided the list of trial websites we examined, as well as those excluded, in Multimedia Appendix 2.

**Table 1:** Summary of articles included in scientific journals.

Study	Name of clinical trial agency	Type of clinical trial agency	Type of regulatory document	Geographical scope	Scientific scope
Nisen and Rockhold, 2013 [72]	GSK^a^-sponsored trials	For-profit pharmaceutical trial agency	Policy	Global	GSK-sponsored trials
Ross et al, 2018 [70]	YODA^b^	Academic	Policy	Not reported	YODA partners (Medtronic and Johnson & Johnson)
Pencina et al, 2016 [73]	SOAR^c^ initiative	For-profit pharmaceutical-academia collaboration	Policy	United States	BMS^d^-sponsored phase I to phase IV trials (trials completed after January 2008)
Mitka, 2015 [71]	IOM^e^	Nonprofit regulatory agencies	Guideline	Not reported	Not reported

^a^GSK: GlaxoSmithKline.

^b^YODA: Yale Open Data Access.

^c^SOAR: Supporting Open Access for Researchers.

^d^BMS: Bristol Myers Squibb.

^e^IOM: Institute of Medicine.

### Summary of Articles Included From Scientific Literature Search

We included perspective papers that summarized specific regulatory documents. The scientific scope of these regulatory documents varied significantly. Of the 4 documents included, 3 (75%) [70,72,73] referred to a specific policy document, whereas 1 (25%) [71] referred to a data-sharing guideline. These documents were published between 2013 and 2018. The trial agencies specified in these documents were a for-profit pharmaceutical firm (GlaxoSmithKline [GSK]-sponsored clinical trials) [72], an academic institution (Yale Open Data Access) [70], a pharmaceutical-academia collaboration (Supporting Open Access for Researchers) [73], and a nonprofit regulatory agency (Institute of Medicine) [71]. The GSK policy is applicable to GSK-sponsored trials globally, whereas the Supporting Open Access for Researchers initiative is applicable to Bristol Myers Squibb (BMS)–sponsored phase I-IV interventional clinical trials completed after 2008 in the United States. The remaining 50% (2/4) of the documents [70,71] did not specify geographical scope. The Yale Open Data Access project policy is applicable to their partner clinical trial agencies such as trial data sponsored by Medtronic and Johnson & Johnson clinical trial data [70] (Table 1).

### Summary of Regulatory Documents Identified Through Gray Literature Search

We report the summary of the included regulatory documents in the gray literature in Table 2. In case of multiple versions, the most recent version of these documents was considered for coding the data. All the clinical trial agencies were from the United States and the United Kingdom. For-profit pharmaceutical trial agencies [41,74] (Celgene, Pharmaceutical Research and Manufacturers of America, and European Federation of Pharmaceutical Industries and Associations) follow regulations applicable to the trials conducted in the United States and the European Union. The NIH [11,16,42-44,75] and the Medical Research Council [76] have a data-sharing policy applicable to their own funded clinical trials. The policies of NIH-affiliated agencies [16,42] are applicable to trials with a grant limit of US $500,000 or more in direct costs in any year of the proposed project period. None of the other policy documents specify a grant limit for their applicability (Table 2).

**Table 2:** Summary of regulatory documents (gray literature).

Policy	Recent policy version	Country of origin	Geographical scope	Scientific scope	Timeline	Grant limit (US $)
Celgene [41]	Version 4, 2017	United States	United States and European Union	Compound and indication trials	—^a^	—
PhRMA^b^ and EFPIA^c^ [74]	2013	United States	United States and European Union	—	—	—
NIH^d^-1 [42]	2003	United States	—	NIH-funded studies	From 2003 to 2023	≥500,000 or more (in direct costs^e^)
NIH-2 [16]	2020	United States	—	NIH-funded or NIH-conducted research	From 2023	≥500,000 or more (in direct costs^e^)
NIH-NHLBI^f^ [43]	2014	United States	—	NHLBI-funded studies (related to heart, lung, and blood–related research)	From 2003 to 2023	—
NIH-NCI^g^ [44]	2016	United States	—	NCI-supported Cancer Moonshot studies	On or after 2017	—
MRC^h^ [76]	2015	United Kingdom	—	Publicly funded clinical trials units	—	—
NIHR^i^ [75]	Version 1, 2019	United Kingdom	—	NIHR-funded research studies	—	—
EMA^j^ [79]	Policy 0070, 2018	United Kingdom	—	For academic and noncommercial research purposes	Data held after July 1, 2015	—
NIH StrokeNet [11]	Version 1, 2014	United States	—	Clinical trials conducted in the NIH StrokeNet network	—	—
PCTU^k^ [10]	Version 5, 2019	United Kingdom	—	Clinical research data held on PCTU servers	From January 1, 2014	—
PCORI^l^ [77]	2018	United States	—	Research projects funded by PCORI	—	—
UKCRC^m^ [78]	2021	United Kingdom	—	UKCRC-registered clinical trials unit network	—	—

^a^Not mentioned.

^b^PhRMA: Pharmaceutical Research and Manufacturers of America.

^c^EFPIA: European Federation of Pharmaceutical Industries and Associations.

^d^NIH: National Institutes of Health.

^e^In any year of the proposed project period through grants, cooperative agreements, or contracts.

^f^NHLBI: National Heart, Lung, and Blood Institute.

^g^NCI: National Cancer Institute.

^h^MRC: Medical Research Council.

^i^NIHR: National Institute for Health Research.

^j^EMA: European Medicines Agency.

^k^PCTU: Pragmatic Clinical Trials Unit.

^l^PCORI: Patient-Centered Outcomes Research Institute.

^m^UKCRC: UK Clinical Research Collaboration.

### Data-Sharing Mechanisms

We reviewed data-sharing mechanisms from the documents and summarize the key specifications in Table 3. Most (11/17, 65%) [10,40-42,70-73,75-78] of the clinical trial agencies require a data-sharing agreement between the data requester and the clinical trial agency. The specific requirements to access data and obligations with good data-sharing principles are highlighted in the data-sharing agreement. We could access data-sharing–agreement templates from 27% (3/11) [10,42,76] of these trial agencies. At these trial agencies, data-sharing agreements between the data requester and clinical trial agency are mutual and nondisclosable in nature. Data-sharing agreements ensure that appropriate data anonymization procedures are followed, thereby minimizing the chance of identifying the study participant. These agreements further prohibit data users from sharing data with third parties. However, the legal actions in cases of noncompliance are not clearly defined. The data request process is facilitated either through a website registration procedure or submission of a data request form. Basic information about the principal investigator of secondary research, key personnel, and the research proposal, which includes project title, scientific abstract, brief project background and statement of project significance, specific aims, research methods, narrative summary, project timeline, dissemination plan, and bibliography, are standard requirements across various trial registries. Data requesters need to mention their proposed process for data management and the process of making the resulting publications available to the public. Not all the trial agencies require an independent review committee. However, an independent review committee would possess the right to decide on sharing the data.

Nearly half (8/17, 47%) [10,40,41,43,44,70,72,75,76] of the trial agencies mentioned that informed consent for data sharing should be included in the broader informed consent for research. However, curiously, none of the policy documents mention that consent is mandated. Of these 8 policy documents, 2 (25%) [40,44,70] make statements to the effect that data-sharing practices should follow the data-sharing statement presented in the broader informed consent.

Of the 17 clinical trial agencies, only 5 (29%) [10,40,70-72,76] specified a general timeline within which data would be shared. Of these 5 agencies, 2 (40%) specified that data would be shared along with the publication, 1 (20%) [76] specified a timeline of 18 months of trial completion, and 1 (20%) [10] specified a timeline of 24 months of trial completion. Of these 5 agencies, 1 (20%) [71] specified a separate timeline for data underlying the results, that is, no later than 6 months from the time of publication, and no later than 18 months from the time of publication for IPD. The time limit to access data was described by 29% (5/17) of the clinical trial agencies: 12 months by 80% (4/5) [10,40,70,72] of these agencies and 7 years by 20% (1/5) [77]. Of the 17 clinical trial agencies, 1 (6%) [75] stated that data access would be granted as per the agreement.

Of the 17 trial agencies, 12 (71%) [10,40-44,70,72,74,75,77-79] provided a sufficient description of their data access model. Of these 12 trial agencies, 10 (83%) [10,40-43,70,72,74,75,77,78] practiced a controlled-access model, whereas 2 (17%) [44,79] followed both open- and controlled-access models based on the type of data. Only 35% (6/17) [16,40,42,43,70,71,76] of the clinical trial agencies specified who is to bear the cost of data sharing. Policies identify varied sources responsible for the cost of data sharing: independent funder [40,70], trial sponsor [76], or the clinical trial agency itself [16,42,43]. The Institute of Medicine, a clinical trial agency, has stated that the cost of data sharing should be shared by the clinical trial sponsor and the secondary data user [71]. Clinical trial agencies such as the NIH [16,42], the NIH-affiliated trial agencies [43], and the Medical Research Council [76] encourage trial investigators to estimate data-sharing expenses in the grant application. Most (14/17, 82%) of the data-sharing policies were applicable to IPD sharing. The other clinical trial data in Table 3 refer to case report forms, protocols, reporting, and analysis plans. The included data-sharing policies varied widely in terms of the terminology used to describe the *type of trial data*. To standardize our interpretation of clinical trial data applicability on the type of clinical trial data, we referred to the definitions given in the policy documents. For example, the NIH 2003 policy applies to underlying research data of the final summary statistics and results [42]. By contrast, the new NIH 2023 policy is applicable to all the scientific factual data that are accepted in the scientific community to validate and replicate research findings [16] (Table 3).

**Table 3:** Key elements of data-sharing mechanisms in regulatory documents.

	Requires data-sharing agreement	Requires review committee	Requires informed consent	Specifies timeline to share data	Specifies time limit to access data	Specifies data-sharing model	Specification on cost of data sharing	Requires sharing of IPD^a^	Specification on sharing of other clinical trial data
GSK^b^-sponsored trials [72]	Yes	Yes	Yes (applicable from 2013)	No	Yes	Yes	No	Yes	Yes
YODA^c^ [70]	Yes	Yes	Yes (applicable from 2014)	No	Yes	Yes	Yes	Yes	Yes
SOAR^d^ initiative	Yes	Yes	No	No	No	Cannot ascertain	No	Yes	Yes
IOM^e^ [71]	Yes	Yes	No	Yes	No	Cannot ascertain	Yes	Yes	Yes
Celgene [41]	Yes	Yes	Yes (applicable from 2014)	No	No	Yes	No	Yes	Yes
PhRMA^f^ and EFPIA^g^ [74]	No	Yes	No	No	No	Yes	No	Yes	Yes
NIH^h^-1 [42]	Yes	Yes	No	No	No	Yes	Yes	No	Yes
NIH-2 [16]	No	No	No	No	No	Cannot ascertain	Yes	Yes	Yes
NIH-NHLBI^i^ [43]	No	No	Yes	Yes	No	Yes	Yes	Yes	No
NIH-NCI^j^ [44]	No	No	Yes (if conducting research would benefit public health)	Yes	No	Yes	No	Yes	No
MRC^k^ [76]	Yes	Yes	Yes	Yes	No	Cannot ascertain	Yes	Yes	No
NIHR^l^ [75]	Yes	Yes	Yes	No	Yes	Yes	No	Yes	Yes
EMA^m^ [79]	No	Yes	No	No	No	Yes	No	Yes	Yes
NIH StrokeNet [11]	No	No	No	No	No	Cannot ascertain	No	No	No
PCTU^n^ [10]	Yes	Yes	Yes	Yes	Yes	Yes	No	Yes	Yes
PCORI^o^ [77]	Yes	Yes	No	No	Yes	Yes	No	Yes	No
UKCRC^p^ [78]	Yes	No	No	No	No	Yes	No	No	No
Total, n (%; yes or no)	11 (65)	12 (71)	8 (47)	5 (29)	5 (29)	12 (71)	6 (35)	14 (82)	11 (65)

^a^IPD: individual participant data.

^b^GSK: GlaxoSmithKline.

^c^YODA: Yale Open Data Access.

^d^SOAR: Supporting Open Access for Researchers.

^e^IOM: Institute of Medicine.

^f^PhRMA: Pharmaceutical Research and Manufacturers of America.

^g^EFPIA: European Federation of Pharmaceutical Industries and Associations.

^h^NIH: National Institutes of Health.

^i^NHLBI: National Heart, Lung, and Blood Institute.

^j^NCI: National Cancer Institute.

^k^MRC: Medical Research Council.

^l^NIHR: National Institute for Health Research.

^m^EMA: European Medicines Agency.

^n^PCTU: Pragmatic Clinical Trials Unit.

^o^PCORI: Patient-Centered Outcomes Research Institute.

^p^UKCRC: UK Clinical Research Collaboration.

## Discussion

### Principal Findings

Data-sharing practices have not been a characteristic of most randomized controlled clinical trials. There may be many reasons for this: for example, incentives to shift the status quo away from proprietary models of holding on to data have often remained diminutive. For producers of data, such as large corporations, the alignment of financial investments dovetails well with the desire to amortize these costs in terms of limiting data sharing to the originators of the data. On the demand side, firms or entities with the technical capacities to use such data are limited to competitor firms in the mostly capitalist Organisation for Economic Co-operation and Development countries. Emerging market firms from Brazil, Russia, India, China, and South Africa, or other nations with relatively more sophisticated technical capacities in reverse engineering are another class of potential consumers of such data. Institutionally, at the international level, the WTO has governed international trade in goods and services since its inception in 1995. As global trade requires abiding by WTO standards, there has been a cross-national harmonizing effect. This affects all goods and services in international trade. Global standards for goods and services have to be followed by all; to a large degree, this overrides national considerations, and therefore data-exclusivity practices are also introduced to harmonize transfers of both goods and the generation of data over the course of trade in services as well. As far as data-exclusivity practices are concerned, these are buttressed by domestic legal frameworks in the wealthy countries that generally initiate such trials. In the early 2000s, the Doha Declaration as well as debates regarding compulsory licensing episodically brought such issues to the fore but without abiding institutional shifts [28].

However, as the COVID-19 pandemic progressed, the lack of robust evidence hampered effective therapeutic and public health interventions, resulting in widespread panic as cases surged. Despite a large number of clinical trials aimed at repurposing existing interventions for managing COVID-19 and several promising drug candidate interventions undergoing clinical trials, the scientific community was unable to collaborate and synergize efforts. It is possible that this was on account of regulatory and policy bottlenecks that hampered clinical trial data sharing. This scoping review was intended to identify regulatory frameworks and policy guidance that support clinical trial data sharing. We included regulatory documents (n=4) from the scientific literature search as well as from the gray literature search (n=13). Our results indicate that clinical trial regulatory frameworks aim to make data available for noncommercial use for researchers. We noted that clinical trial agencies lack comprehensive approaches that facilitate data sharing. Of the 17 regulatory documents reviewed, 11 (65%) mandate the need for a data-sharing agreement, 8 (47%) require informed consent, 12 (71%) mandate a proposal review committee to oversee the data sharing, and 5 (29%) specify timelines for data sharing and a time limit to access data. A significant proportion (12/17, 71%) of these documents describe different data-sharing models: 83% (10/12) were related to IPD sharing and 33% (4/12) provided specifications regarding the cost of sharing data.

Data sharing is widely advocated as a norm in clinical research. However, regulatory frameworks and policy guidance to support researchers and institutions to share clinical trial data continue to lag behind such norms. This gap between the intention to share data and the lack of supportive regulatory and policy frameworks can be attributed to the direct or indirect effects connected with data sharing. At a macrolevel, this could be due to issues pertaining to IPR, differences across regulatory guidelines in high-income countries and LMICs, the variations in commercial interests of funding sources, and the potentially high economic benefits from data exclusivity [80-82]. Besides these issues, researchers are concerned about ensuring the privacy and confidentiality of study participants; although the informed consent procedures have provisions to seek participants’ consent for data sharing and secondary use of their data, these are rarely implemented in practice. In practice, institutional ethics review boards often resort to myopic approaches when approving clinical trials that propose broader informed consent for data sharing and secondary use of data. These could also hamper the efforts of clinical trial investigators seeking to incorporate specific data-sharing clauses in the informed consent procedures. Moreover, the cost associated with data sharing, potential threats to confidentiality, academic credit, and investigator capacity to standardize data in a shareable manner are some concerns from a researcher’s perspective [80-82]. Our gray literature screening of clinical trial websites showed that not every clinical trial agency is guided by its own data-sharing regulatory document (Multimedia Appendix 2). We noted that policies for clinical trial data sharing are evolving. For instance, the UK Clinical Research Collaboration published a standard operating procedure in 2021 to share participant data [78]; however, it is still at the development stage. Similarly, GSK recently agreed to share deidentified participant data [72].

If present, robust data-sharing practices often come into play when there is a legal agreement between the data requester and the data-sharing agency. The regulatory areas involved in the data-sharing mechanism guide investigators to share data in an appropriate manner to protect participant autonomy and data confidentiality. However, a formal agreement between a data requester and the trial agency is often not outlined in the data-sharing regulatory documents identified in this review. Existing regulatory and policy documents suggest that open access to clinical trial data may not be reliable because of higher chances of fraudulent reports or erroneous secondary analyses. Given that clinical trial participants are often from multiple sites across the world, anonymization practices for the data must meet the respective countries’ regulatory requirements. In the policies we reviewed, the details of cost of data sharing for the infrastructure and maintenance, data standardization, harmonization of data, and data quality assurance have not been described, let alone specified. Up-front resource investments for building sustainable and comprehensive data-sharing platforms with standardized data elements and user-friendly interfaces are likely to enhance the quality, accessibility, and usability of shared data. However, these may be expensive and financially untenable in LMICs. Of the 17 policies, only 6 (35%) [16,40,42,43,70,71,76] mentioned the cost of data sharing. Core clinical trial sponsors and agencies such as the NIH have recognized the need for supporting investigators for data sharing. The NIH states that in grant applications, data-sharing expenses can be estimated separately [16,42]. Such cost sharing is identified as one of the sustainable ways to achieve data sharing and was advocated at a public workshop conducted by the National Academies of Sciences, Engineering, and Medicine in November 2019 [32]. The Institute of Medicine is the only entity with a policy that highlighted the sharing of cost by the sponsor and data user [71].

Of the 17 clinical trial agencies, 5 (29%) [10,43,44,71,76] specified timelines to share data and these specifications referred to trial completion [10,71,76] or publication [43,44] as the milestones for sharing data appropriately. The benefits of data sharing can be maximized when it is encouraged at almost all stages of clinical trials; however, specifications regarding data sharing across major stages of clinical trials are often missing [83]. Furthermore, we found that none of the regulatory documents specified incentives or any kind of reward for data sharing. In addition, specifications relating to noncompliance with regard to data sharing are not clear. Data sharing from large multicenter international clinical trials is challenging because of the varied practices as guided by the respective national regulatory bodies [84]. We found that informed consent and legal agreements for data access are not a requirement for all policies. The regulatory frameworks do not cover all key elements of the data-sharing mechanisms discussed in this scoping review. However, it is encouraging to see the scope of these policies covering IPD sharing, rather than limiting sharing to overall clinical trial data.

The goal of any research involving human participants is to improve the health and quality of life of humans. Therefore, it is the need of the hour to look at data sharing with a moral and ethical lens for the public good. It is important to weigh the risks against the benefits of data sharing and find ways to overcome or mitigate the risks. Any data-sharing attempt without considering the trial funders is unlikely to be successful. Data sharing requires collaboration among apex federal or national trial agencies, academic institutions, and profit-based pharmaceutical clinical trial agencies. The cost of sharing data is another unexplored area that needs to be addressed. Either the main trial agency or another trial funder can play an important role in providing financial assistance and the capacity building of investigators for data sharing. A comprehensive data-sharing policy may not be feasible, given the diverse approaches in clinical research, geographies, and the scientific merit of a given clinical trial. Regulatory frameworks need to acknowledge these factors when standardizing data-sharing processes and provide a clear description for trial investigators rather than a broader document in support of data sharing. There is a strong need to define policy scope in terms of type of clinical trial, type of clinical trial data, and single-center or multicenter (including multicountry) trials, as well as specifications for privately funded and publicly funded trials or commercial and noncommercial funders. A clear distinction between *mandated requirements* and *supportive requirements* during the data-sharing process would help investigators to practice data sharing. To provide better participant privacy and confidentiality, there should be a neutral party to check the information in the data set. Creating an independent review panel to decide on the accessibility of the clinical data should be encouraged. A systematic review on increasing data sharing in health and medical research showed that there is a lack of research on evidence-based data sharing [85]. Nurturing clinical trial investigators, clinical trial funders, and academicians with rewards for data sharing should be encouraged. Data sharing from clinical trials is a daunting task; nevertheless, it is important to make this process easy for researchers, university academics, clinical trial agencies, and funders considering the benefits of data sharing. Ensuring viable, efficient, and feasible data-sharing mechanisms tailored to stakeholders’ interests and bound to ethical and legal concerns is the way forward.

### Strengths and Limitations

This scoping review used a systematic, replicable, and rigorous approach to summarize evidence. By using well-defined search terms, database searches, and screening of articles, our processes were rigorous because these were carried out independently by 2 authors (NG and SP or PK and TC). We carried out an extensive gray literature search and reference screening of the articles that were included at the full-text screening stage. Although stakeholders were contacted while finalizing the objectives, we were unable to consult them while drafting the results because of the nature of the review, and we would like to acknowledge this as a limitation. We had initially planned to carry out searches in scientific databases but later decided to perform the gray literature search, and this is reported as a deviation from our protocol.

### Conclusions

This scoping review used a rigorous methodology to support clinical trial data sharing. Standardizing data-sharing processes by framing a more focused and concise policy with key elements of data-sharing mechanisms could be feasible and easier to practice than a single, rigid, and comprehensive data-sharing policy. We believe that to uncover the complexities and make data sharing a reality for the public good, negotiations around stakeholder interests are crucial. During and after the COVID-19 pandemic, and to paraphrase what Victor Hugo once remarked in another context, clinical data sharing may well be “an idea whose time has come.”

## Appendix

Multimedia Appendix 1 Search strategy.

Multimedia Appendix 2 Gray literature search.

Multimedia Appendix 3 Data-coding template.

## Abbreviations

GSK: GlaxoSmithKline
IPD: individual participant data
IPR: intellectual property rights
LMIC: low- and middle-income country
NIH: National Institutes of Health
PRISMA: Preferred Reporting Items for Systematic Reviews and Meta-Analyses
PRISMA-ScR: Preferred Reporting Items for Systematic Reviews and Meta-Analyses extension for Scoping Reviews
WTO: World Trade Organization
