# Supplementary material for: Regulatory Frameworks for Clinical Trial Data Sharing: Scoping Review
Source: J Med Internet Res. 2022 May 4;24(5):e33591. doi: 10.2196/33591 (PMC9118011; doi:10.2196/33591)
Supplement: Multimedia Appendix 1 [file jmir_v24i5e33591_app1.docx]

**Multimedia Appendix 1.** Search strategy.

| Database | Date | Search strategy | Number of articles |
| --- | --- | --- | --- |
| PubMed | 11/05/ 2021 | ((("Guidelines as Topic"[Mesh] OR "Practice Guidelines as Topic"[Mesh]) OR ((((((((((guideline) OR (guidelines)) OR (policy)) OR (policies)) OR (regulation)) OR (regulations)) OR (framework)) OR (frameworks)) OR (SOP)) OR ("standard operating procedure"))) AND (((("clinical trial data") OR ("individual patient data")) OR ("individual participant data")) OR (IPD))) AND ((("Access to Information"[Mesh]) OR ("Information Dissemination"[Mesh])) OR (((sharing) OR (exchange)) OR (dissemination))) | 443 |
| Web of science | 11/05/ 2021 | #1 TI=(sharing OR access OR dissemination) OR TS=(sharing OR access OR dissemination) OR AB=(sharing OR access OR dissemination)  #2 TS=("clinical trial data" OR "individual patient data" OR "individual participant data" OR IPD) OR AB=("clinical trial data" OR "individual patient data" OR "individual participant data" OR IPD)  #3  TS=(guideline OR guidelines OR policy OR policies OR regulation OR regulations OR framework OR frameworks OR SOP OR "standard operating procedure") OR AB=(guideline OR guidelines OR policy OR policies OR regulation OR regulations OR framework OR frameworks OR SOP OR "standard operating procedure")  #3 AND #2 AND #1  *Databases= WOS, KJD, RSCI, SCIELO Timespan=2000-2021*  *Search language=English* | 291 |
| Scopus | 11/05/21 | TITLE-ABS-KEY ( guideline  OR  guidelines  OR  policy  OR  policies  OR  regulation  OR  regulations  OR  framework  OR  frameworks  OR  sop  OR  "standard operating procedure" )  AND  TITLE-ABS-KEY ( "clinical trial data"  OR  "individual patient data"  OR  "individual participant data"  OR  ipd )  AND  TITLE-ABS-KEY ( sharing  OR  exchange  OR  dissemination )  AND  ( LIMIT-TO ( LANGUAGE ,  "English" ) ) | 226 |
| Embase | 11/05/ 2021 | ('standard operating procedure' OR guideline OR guidelines OR policy OR policies OR regulation OR regulations OR framework OR frameworks OR sop) AND ('clinical trial data':ti,ab,kw OR 'individual patient data':ti,ab,kw OR 'individual participant data':ti,ab,kw OR ipd:ti,ab,kw) AND (sharing:ti,ab,kw OR exchange:ti,ab,kw OR dissemination:ti,ab,kw) AND english:la AND [2000-2021]/py | 191 |
| ProQuest | 11/05/2021 | (Exact("Guidelines as Topic" OR "Guidelines as Topic; Humans" OR "Practice Guidelines as Topic") OR (guideline) OR (guidelines) OR (policy) OR (policies) OR (regulation) OR (regulations) OR (framework) OR (frameworks) OR (SOP) OR ("standard operating procedure")) AND (("clinical trial data") OR ("individual patient data") OR ("individual participant data") OR (IPD)) AND (Exact("Access to Information" OR "Information Dissemination") OR (sharing) OR (exchange) OR (dissemination)) | 67 |
