# Supplementary material for: Regulatory Frameworks for Clinical Trial Data Sharing: Scoping Review
Source: J Med Internet Res. 2022 May 4;24(5):e33591. doi: 10.2196/33591 (PMC9118011; doi:10.2196/33591)
Supplement: Multimedia Appendix 2 [file jmir_v24i5e33591_app2.docx]

**Multimedia Appendix 2.** Gray literature search.

| Policy/trial agency name | Decision | Reason for exclusion |
| --- | --- | --- |
| European Clinical Research Infrastructure Network [1] | Exclusion | No policy/guideline found |
| US Department of Veterans Affairs [2] | Exclusion | No policy/guideline found |
| SRDR Data Sharing Policy [3] | Exclusion | Policy scope: Systematic review |
| American Heart Association [4] | Exclusion | No policy/guideline found |
| WHO Joint Statement Common Policy Elements [5] | Exclusion | Policy scope: Sharing trial results |
| Govt of UK [6] | Exclusion | No policy/guideline found |
| Clinical Study Data Request [7] | Exclusion | Policy scope: Data repository |
| World-Wide Antimalarial Resistance Network [8] | Exclusion | Policy scope: Data repository |
| Food and Drug Administration [9] | Exclusion | No policy/guideline found |
| International Committee of Medical Journal Editors [10] | Exclusion | Policy scope: Academic journals |
| Research Data Alliance [11] | Exclusion | Policy scope: Data repository |
| Project Data Sphere [12] | Exclusion | Policy scope: Data repository |
| National Institute of Allergy and Infectious Diseases [13] | Exclusion | No policy/guideline found |
| European Medicines Agency [14] | Inclusion |  |
| Celgene [15] | Inclusion |  |
| Pharmaceutical Research and Manufacturers of America [16] | Inclusion |  |
| National Institutes of Health (NIH)- 2003 [17] | Inclusion |  |
| National Institutes of Health- 2023 [18] (Effective from 25 January 2023) | Inclusion |  |
| NIH- National Heart, Lung, and Blood Institute [19] | Inclusion |  |
| NIH- Cancer Moonshot [20] | Inclusion |  |
| Medical Research Council [21] | Inclusion |  |
| National Institute for Health Research [22] | Inclusion |  |
| Pragmatic Clinical Trials Unit [23] | Inclusion |  |
| Yale University Open Data Access project policy [24] | Inclusion |  |
| Patient-Centered Outcomes Research Institute [25] | Inclusion |  |
| United Kingdom-Clinical Research Collaboration [26] | Inclusion |  |

References

1. European Clinical Research Infrastructure Network. Available from: <https://ecrin.org/>. Accessed 18 Aug 2021.
2. US Department of Veterans Affairs. Available from <https://www.va.gov//>. Accessed 18 Aug 2021.
3. SRDR Data Sharing Policy. Available from <https://srdr.ahrq.gov/home/policies>. Accessed 18 Aug 2021.
4. American Heart Association Available from <https://www.heart.org/>. Accessed 18 Aug 2021.
5. World health organization. Joint statement on public disclosure of clinical trials. Available from <https://www.who.int/news/item/18-05-2017-joint-statement-on-registration>. Accessed 18 Aug 2021.
6. GOV UK. The Medicines and Healthcare products Regulatory Agency. Available from: <https://www.gov.uk/government/organisations/medicines-and-healthcare-products-regulatory-agency>. Accessed 18 Aug 2021.
7. Clinical Study Data Request. Available from: <https://www.clinicalstudydatarequest.com/>. Accessed 18 Aug 2021
8. World-Wide Antimalarial Resistance Network. Available from <https://www.wwarn.org/>. Accessed 18 Aug 2021.
9. Food and Drug Administration. Available from: <https://www.fda.gov/>. Accessed 18 Aug 2021.
10. International Committee of Medical Journal Editors. Available from: <http://www.icmje.org/news-and-editorials/data_sharing_june_2017.pdf>. Accessed 18 Aug 2021.
11. Research Data Alliance. Available from: <https://www.rd-alliance.org/group/rda-covid19-rda-covid19-omics-rda-covid19-epidemiology-rda-covid19-clinical-rda-covid19-1>. Accessed 18 Aug 2021.
12. Project Data Sphere. Available from: <https://www.projectdatasphere.org/about/data-sharing-philosophy>. Accessed 18 Aug 2021.
13. National Institute of Allergy and Infectious Diseases. Available from: <https://www.niaid.nih.gov/>. Accessed 18 Aug 2021.
14. European Medicines Agency. Available from: <https://www.ema.europa.eu/en/documents/regulatory-procedural-guideline/external-guidance-implementation-european-medicines-agency-policy-publication-clinical-data_en-1.pdf>. Accessed 18 Aug 2021.
15. Celgene. Available from: <https://www.celgene.com/research-development/clinical-trials/clinical-trials-data-sharing/>. Accessed 18 Aug 2021
16. Pharmaceutical Research and Manufacturers of America. Available from: <https://www.phrma.org/en/Codes-and-guidelines/PhRMA-Principles-for-Responsible-Clinical-Trial-Data-Sharing-Certifications>. Accessed on 18 Aug 2021.
17. National Institutes of Health. Available from: <https://grants.nih.gov/grants/policy/data_sharing/data_sharing_guidance.htm>. Accessed 18 Aug 2021.
18. National Institutes of Health. Available from: <https://grants.nih.gov/grants/guide/notice-files/NOT-OD-21-013.html>. Accessed 18 Aug 2021.
19. National Heart, Lung, and Blood Institute. Available from: <https://www.nhlbi.nih.gov/grants-and-training/policies-and-guidelines/nhlbi-policy-for-data-sharing-from-clinical-trials-and-epidemiological-studies>. Accessed 18 Aug 2021.
20. National Institute of Cancer. Available from: <https://www.cancer.gov/research/key-initiatives/moonshot-cancer-initiative/funding/public-access-policy#definitions>. Accessed 18 Aug 2021.
21. Medical Research Council. Available from: <https://mrc.ukri.org/research/policies-and-guidance-for-researchers/open-research-data-clinical-trials-and-public-health-interventions/>. Accessed 18 Aug 2021.
22. National Institute for Health Research. Available from: <https://www.nihr.ac.uk/about-us/document-library.htm?custom_in_type=9697>. Accessed 18 Aug 2021.
23. Pragmatic Clinical Trials Unit. Available from: <https://www.qmul.ac.uk/pctu/>. Accessed 18 Aug 2021.
24. Yale University Open Data Access project policy. Available from: <https://yoda.yale.edu/policies-procedures-guide-external-investigator-access-clinical-trial-data>. Accessed 18 Aug 2021.
25. Patient-Centered Outcomes Research Institute. Available from: <https://www.pcori.org/search/site?keyword=data%20sharing%20policy>. Accessed 18 Aug 2021.
26. United Kingdom-Clinical Research Collaboration. Available from: <https://cdn.ymaws.com/www.ukcrc-ctu.org.uk/resource/resmgr/5_year_strategy/network_5_year_strategy_2019.pdf>. Accessed 18 Aug 2021.
