# Supplementary material for: Regulatory Frameworks for Clinical Trial Data Sharing: Scoping Review
Source: J Med Internet Res. 2022 May 4;24(5):e33591. doi: 10.2196/33591 (PMC9118011; doi:10.2196/33591)
Supplement: Multimedia Appendix 3 [file jmir_v24i5e33591_app3.docx]

**Multimedia Appendix 3.** Data-coding template.

| SL No | Domains | Variables |
| --- | --- | --- |
| Data extraction template for scientific literature | | |
| 1 | Article identifiers | PMID |
|  |  | Title |
|  |  | First author |
|  |  | Country affiliation of first author |
|  |  | Country of corresponding author |
|  |  | Year of publication |
|  |  | Journal name |
| 2 | Type of literature | Editorial/Commentary/Policy brief |
| 3 | Name of referred regulatory agency |  |
| 4 | Type of trial agency | Pharmaceutical-based/federal regulatory agencies/academic/non-profit research organization/ collaborative network of CTUs |
| 5 | Policy scope | Geographical |
|  |  | Scientific scope |
|  |  | Grant limit |
|  |  | Data type |
| 6 | Data sharing mechanism | Timeline to share data |
|  |  | Timeline for data availability |
|  |  | Mode of data sharing |
|  |  | Cost of data sharing |
|  |  | Data sharing agreement and specifications |
|  |  | Informed consent for data sharing |
|  |  | Data request process |
|  |  | Data access model |
|  |  | Review of data request proposals and review committees |
| Data extraction template form Grey literature | | |
| 1 | Policy identifiers | Agency name |
|  |  | Document title |
|  |  | Website link |
|  |  | Recent policy revision number |
|  |  | Year of recent revision |
|  |  | Primary/original country |
|  |  | Type of regulatory document |
| 2 | Type of trial agency | Pharmaceutical-based/federal regulatory agencies/academic/non-profit research organization/ collaborative network of CTUs |
| 3 | Policy scope | Geography |
|  |  | Timeline |
|  |  | Scientific scope |
|  |  | Grant limit |
|  |  | Data type |
| 4 | Data sharing mechanism | Timeline to share data |
|  |  | Timeline for data availability |
|  |  | Mode of data sharing |
|  |  | Cost of data sharing |
|  |  | Data sharing agreement and specifications |
|  |  | Informed consent for data sharing |
|  |  | Data request process |
|  |  | Data access model |
|  |  | Review of data request proposals and review committees |
